# The design and implementation of the software tracking cervical and lumbar vertebrae in spinal fluoroscopy images

**DOI:** 10.4155/fsoa-2017-0089

**Published:** 2017-09-11

**Authors:** Behrouz Alizadeh Savareh, Yousef Sadat, Azadeh Bashiri, Mehraban Shahi, Nasrin Davaridolatabadi

**Affiliations:** 1 Student Research Committee, School of Allied Medicine, Shahid Beheshti University of Medical Sciences, Tehran, Iran; 2 Department of Health Information Management, School of Allied Medical Sciences, Shahid Beheshti University of Medical Sciences, Tehran, Iran; 3 Department of Health Information Management, School of Allied Medical Sciences, Tehran University of Medical Sciences, Tehran, Iran; 4 Department of Health Information Management, Faculty of Para-Medicine, Hormozgan University of Medical Sciences, Bandar Abbas, Iran

**Keywords:** design, frobin, implementation, physiotherapy, software

## Abstract

**Aim:**

Manual analysis of neck kinematics is usually associated with measurement errors and it requires the use of software capabilities. Considering laboratory usage, software has been developed to solve the associated problems.

**Materials & methods:**

Fluoroscopic images taken from 78 women were used to design and evaluate the performance of the software. The software was implemented using C# language, according to the case-based reasoning technique.

**Results:**

The viewpoints of experts suggest accuracy of the software in tracking and calculations, which meets their information requirements.

**Conclusion:**

Using the software could help physiotherapists to accomplish their work in decreased time and with improved accuracy.

## Lay abstract

Manual analysis of neck kinematics usually suffers from measurement errors and requires the use of the software capabilities. In this research article, a software has been developed to solve this problem. The software is implemented using C# language, according to the case-based reasoning technique. Experts using the software suggested its accuracy in tracking and calculations, which met their information requirements. Using the software could help physiotherapists to accomplish their work in decreased time and improved accuracy. However, further research is to confirm these results.

Lumbar pain, a significant problem of public health in industrialized countries is considered as one of the main causes of functional limitations in adults [1]. Neck pain is also one of the most common musculoskeletal damages, and 33–54% of people experience neck pain throughout their life. Furthermore, 14–32% of people in society suffer from chronic neck pain [2]. If the kinematics of the spine is impaired due to pain or limited range of motion, the abnormal intervertebral movements may lead to the onset of clinical symptoms in patients [3,4]. In such a situation, the assessment of the intervertebral movements can provide essential information for the clinical diagnosis of the dysfunction of cervical spine [5]. Identifying and tracking specific spots on the vertebrae in radiographic or fluoroscopic images is considered as an accurate method to measure the quantitative intervertebral movements [6–8]. Accurate examination of translational movements of the vertebrae of the spine during the movement of the neck, especially on a sagittal plane, helps us to better understand biomechanics and treat the dysfunction of spinal joints. The assessment of rotation shows the range of the motion of vertebrae at the sagittal plane of the neck. In addition, it helps to measure the overall dynamic of the cervical spine and test the manual and surgical therapies [6,9–11]. The movements of cervical vertebrae and the contribution of each vertebra in a full range of motion is one of the most important kinematic variables. Examining the motion path of the instant center of rotation is of clinical significance because it can be helpful in diagnosing deviations or abnormalities of normal segmental motion in a sagittal plane [8].

The development of new technologies facilitates the procedures in many areas including the applications of medical diagnosis [12] such as software used in medical image processing [13]. A variety of software products having the capability of computer-aided detection can help experts through automating the process of the interpretation of medical images [14]. In this regard, various software-based methods have been used to analyze the intervertebral movements. Frobin is one of the most important methods in which the height of individuals and the size of intervertebral movements are modified in terms of radiographic magnification. In this method, the measured movements are neither under the influence of x-ray deflection nor the error due to deviation in patient position during imaging [7,15]. Furthermore, in this method, the defined indicators, their parameters, rotation and translation are not quite under the influence of the individual errors and the x-ray deflection [7]. Frobin requires great accuracy; hence one of the most common errors in measuring intervertebral movements of the spine is the measurement error in the rotation and translation of the vertebrae by the examiner manually [15]. To solve this problem, the creation process of software for tracking vertebrae and Frobin calculation are described in this study.

## Method

A set of fluoroscopic images of the neck related to the evaluation of the women’s posture was used to evaluate the performance of the software. As mentioned in [16], a convenient sample of 78 healthy females aged 20–32 years (mean 23 ± 2.63) participated in analytic observational experiment. In that study, subjects moved their head and neck into flexion and extension in the full range and gradually reduced the range of motion to cease movement and maintained the head and neck in the participant’s natural position.

**Figure 1.**
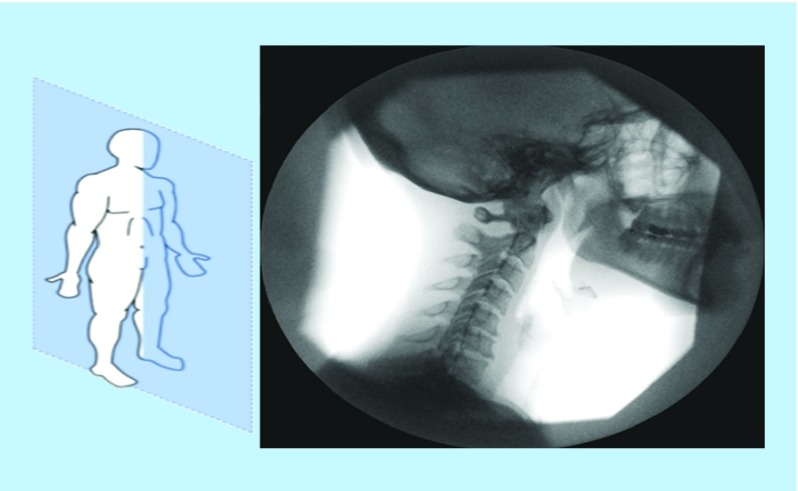
**Vertebra images in sagittal plane.**

The images include a series of fluoroscopic images used to evaluate the performance of the software. A digital imaging technique was used to evaluate head and neck posture in the standing position. A digital camera was placed at a distance of 1.5 m on a fixed point without rotation or tilt. The imaging process was led to a series of neck vertebrae images in sagittal plane as in Figure 1.

**Figure 2.**
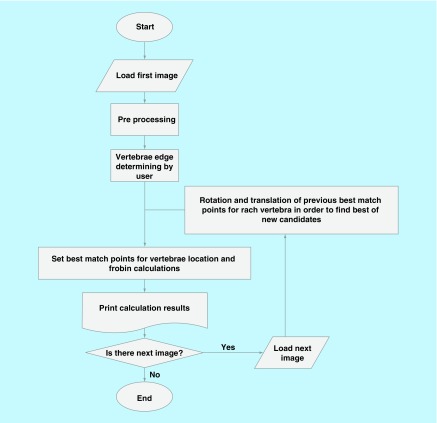
**The general process of software execution.**

According to the aim of actual use for software out of the lab, C# programming language in visual studio and the Aforge website were used to implement the software [17]. Aforge is an open-source software framework of C# designed to be used in the field of computer vision and artificial intelligence. It includes a set of libraries and sample codes to perform a variety of related processes [18]. Using the capabilities of C#, the software was designed in an object-oriented form and all the concepts such as vertebra, spot, line, bisector, angle, etc., and concepts such as digital filters, Frobin calculation and other processing were modeled as object oriented modeling. The software is used as a combination of modules to read, process images, apply filters, print, display output and for Frobin calculations. The general process of software execution is shown in Figure 2.

The steps taken during the software execution are explained in the rest of this section.

### Preprocessing

First, contrast normalization and histogram equalization were used to increase the quality of images. As edge detection is one of the main processes of object detection in many image processing applications [19], using Canny filter, the edges of the vertebrae are determined to a desirable form. After the edge detection, gray images are converted to binary images based on the thresholds. As a result, the edges of the rest of the image are distinguished [19,20].

### Finding the location of the vertebrae

At this point it is necessary to explain the concepts presented. A vertebra is in rotational motion when it is displaced in a spinning or angular displacement around sagittal plane. Also a vertebra is in translational motion when all reference structures indicated within it have the same direction of motion relative to a fixed point in sagittal plane. As already mentioned, the main challenge is to identify the location of vertebrae in the images and track them. In the first image, the location of each vertebra is identified by drawing outer contours of vertebrae by an expert. Using edge detection before this step helps the expert to draw more accurate contours for vertebrae which helps the expert by drawing the contours only on the extracted edges. The software controls the probable vibration of the expert hand and refrains from plotting the line outside the specified edges. The input by expert in the first image forms the foundation of next processing steps, since all subsequent calculations are based on the information that is entered in the first step.

To avoid the complexity of the implementation, case-based reasoning is selected. Its main idea is that the similar issues have the similar responses [21], based on the vertebra location in the first image, translation and rotation calculated. Then, finding the highest amount of their translational and rotational coincidence with next images, the new location of the vertebra is identified. If finding the location of each vertebra in every image is considered as a new question in case-based reasoning, the next question is answered by translations and rotations on the vertebra location in the previous question response. Measuring the coincidence is based on Equation 1:
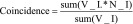



Equation 1. Coincidence equation.

N_I: Next image; V_I: Vertebrae section image.

During three nested loops, the changes of rotation as well as vertical and horizontal translation are made to the previous location of the vertebrae and coincidences are calculated. The next location of the vertebra is identified, based on the highest amount of coincidence. Since the locations of the vertebrae are determined by experts in the first image and all the next images obtain the required information from the previous image, the process is repeated for the next images until all the images are examined.

#### Calculation

**Figure 3.**
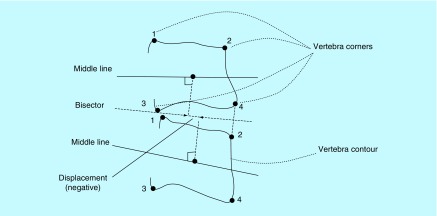
**Calculating displacement between upper and lower vertebrae.**

After extracting the location of each vertebra, the data are fed into the calculation section. Based on Frobin, movement is calculated as follows:

Based on the four corners set by the expert, the geometric center of the determined vertebra and the middle plane of the adjacent vertebra are drawn. Next, the bisector of the angle between the middle screens of the two adjacent vertebrae is drawn. To calculate the movement (between two adjacent vertebrae), a perpendicular line is drawn from their center to the above mentioned bisector. The movement is also calculated based on the distance between the intersections of the two perpendicular lines on the bisector. If the intersection of the perpendicular line of the upper vertebra is ahead of the intersection of the vertical line of the lower vertebra on the bisector line, the movement is forward and is marked by a positive sign. If the intersection of the perpendicular line of the upper vertebra on the bisector line is at the back of the intersection of the perpendicular line of the lower vertebra, the movement is backward and the translation is marked with a minus sign (Figure 3).

The Cobb angle is calculated as a measure for the curvature of the spine in the following manner:

- Drawing the midline of the upper vertebra based on the position of the corners of the upper vertebra;
- Drawing the midline of the lower vertebra based on the position of the corners of the lower vertebra;
- Determining geometric center of each vertebral body as the intersection of the line connecting corner 1 and 4 and the line connecting corner 2 and 3;
- Drawing a perpendicular line to each of the upper lines;
- Investigating the intersection of the two vertical lines and calculating the angle formed by the two lines [22].

### Output production

After scanning an image, extracting the location of the vertebrae and performing calculation, this section creates a text file and saves it in a defined path. The file includes the translation and rotation of each vertebra relative to its previous location and to the location of the upper and lower vertebrae. It is the main output of the software in text form and helps experts to analyze the patient’s condition. In addition, a series of colored images (each color for a tracked vertebra on a binary image) are saved on the specified path.

### Assessing the validity of the software

Given the fact that the software was created for the purpose of actual use by professionals to test the software, it was necessary to use expert opinions to evaluate it. In order to assess the performance of the software in terms of accuracy, five physiotherapists who were involved in the treatment of motor neck problems were selected in an accessible manner. They were provided with the software package (installation files) and asked to work with the software for a month and conduct the analyses of tracking the vertebrae, and finally give their comments and feedback about its performance in text format containing comments qualitatively about the performance of the software and its accuracy in performing the assigned tasks.

## Results

**Figure 4.**
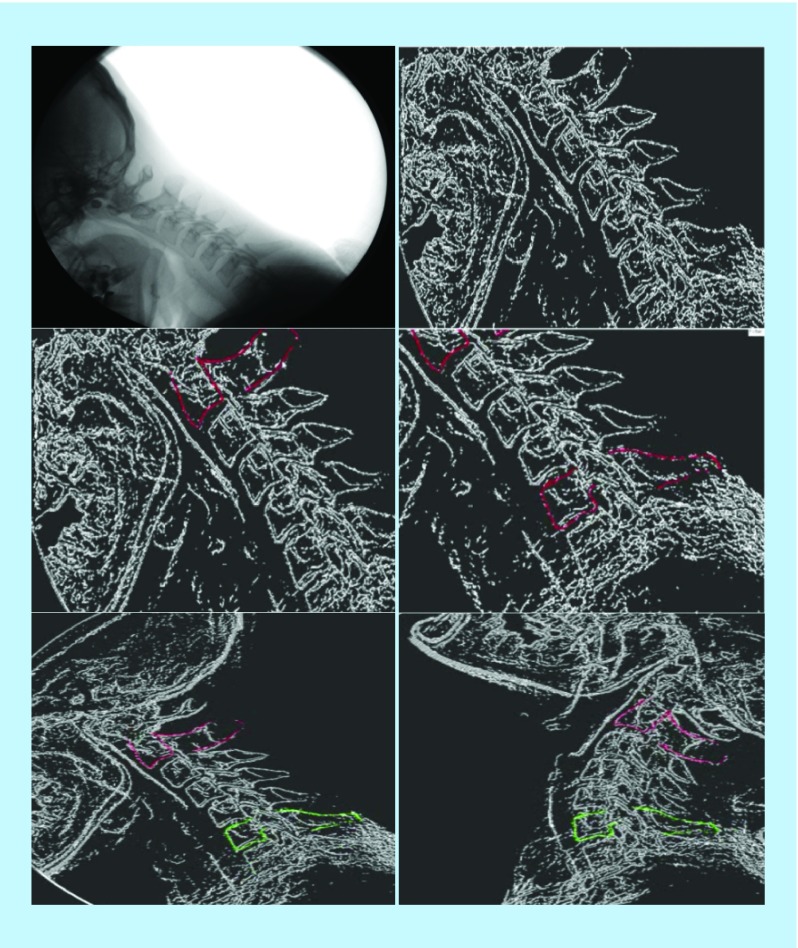
**The overall process of software operation.**

As the software is designed based on image-processing procedure, a process with a number of steps on images took place to produce the desired output. Figure 4 shows the process performed on a sample image.

As shown in Figure 4, the process was carried out on the image (on top-left: original image; on top-right: converting the gray image to binary image; in the middle-left: highlighting one of the vertebrae by an expert; in the middle-right: highlighting the second vertebra by the expert; at the bottom-left: starting the automatic process of tracking in one of the original images; at the bottom-right: continuing the automatic tracking of vertebrae and reaching the final image).

The viewpoints of the experts on assessing the performance of the software indicate that they were satisfied with the performance of the software, and they reported that the location of the vertebrae was identified accurately. Additionally, the colored images of the location of the vertebrae (as shown in Figure 4) at the bottom (left and right) of the image helped them to assess the performance of the software. They are used to ensure the proper functioning of the software and confirm the performed calculation. The reports produced by the software were in the form of text files, according to the viewpoints of the experts. Moreover, they were based on their information requirements and can be used for a variety of clinical diagnoses.

Previously, the analysis of vertebral images was carried out manually and analysis of the vertebral images for each patient which contained more than 100 fluoroscopic images occupied several hours for a specialist. The introduced software decreased the required time for analysis of fluoroscopic images to about 1 h, which is significantly lower than manual and there is no need to interfere with a human agent except for the initial settings. Ease of installation and use were another benefits of the software according to the experts.

## Discussion

In the present study, the procedures of designing practical software for automatic tracking of cervical and lumbar vertebrae in fluoroscopy were described.

Many studies have presented methods of machine learning in vertebrae tracking. A local measure of phase symmetry was used for tracking based on Log–Gabor wavelet transforms in [23]. Furthermore [24], used an automated template-matching algorithm and derivative operators. In addition, iterative estimation approach is used to determine energy function based on complex wavelets in order in [25]. Despite the significant success of these algorithms in the use of modern processing techniques, and the use of C# in implementation, there were limitations in the use of machine learning techniques. Hence, its implementation is carried out with the logic of simplifying the implementation.

The software usability out of the lab environment is the main focus for implementing this software, which is a solution to the problems of the experts in analyzing fluoroscopic images of the spine. It facilitates the process of the diagnosis and treatment of the relevant diseases. One of the main challenges in designing the software is the limitation of the applicable algorithms with regard to the capabilities of C# programming language. Using C# language increases the public usability of the software and it can be easily executed in windows-based platforms. Another benefit of this software is that user interaction and the manipulation of the settings is only in the first image, so tracking and performing calculation are done automatically on the next images. However, in this study, according to the viewpoint of the experts about the accuracy of the software, it is clear that the software meets the information requirements of the experts. Considering the execution procedure of the software, it can be used in other similar medical applications, and fields requiring object tracking and accurate calculation. Slight changes can convert the software as an appropriate tool in similar image processing domains.

## Conclusion & future perspective

In this study, the development of a software for vertebral tracing in fluoroscopic images was described. The evaluation of this software was based on expert opinions. It should be noted that based on the results of future studies and quantitative analysis as a more accurate evaluation of its performance, reliable use of the software in clinical trials could be done. Furthermore, considering the execution procedure of the software, it could also be used in other similar medical applications, such as fields requiring object tracking and accurate calculation. Slight changes can convert the software as an appropriate tool in similar image processing domains. We hope that the current study guides researchers and health professionals to use this software in solving other medical problems requiring the tracing of images and related calculations.

**Summary points**

**Background**

- This study presents the details of a software implementation for tracking cervical and lumbar vertebrae in fluoroscopic images and performing the related calculations.

**Materials & methods**

- The software was developed based on the Frobin method. Its implementation was done using C# language and Aforge.net based on case-based reasoning technique.

**Results**

- The opinions of experts who used the software to do their daily work demonstrate the success of the implementation and the software was able to respond to their information needs.

**Discussion**

- Considering the execution procedure of the software, it could be used in other similar medical applications, fields requiring object tracking and accurate calculation. Slight changes can convert the software as an appropriate tool in similar image processing domains.
